# Detection of Insulin in Insulin-Deficient Islets of Patients with Type 1 Diabetes

**DOI:** 10.3390/life15010125

**Published:** 2025-01-19

**Authors:** Yuliya Krivova, Alexandra Proshchina, Dmitry Otlyga, Anastasia Kharlamova, Sergey Saveliev

**Affiliations:** Laboratory of Nervous System Development, Avtsyn Research Institute of Human Morphology of Federal State Budgetary Scientific Institution “Petrovsky National Research Centre of Surgery”, Tsurupi Street, 3, 117418 Moscow, Russia; proshchina@yandex.ru (A.P.); otlyga@bk.ru (D.O.); grossulyar@gmail.com (A.K.); brainmicroscopy@yandex.ru (S.S.)

**Keywords:** human pancreas, type 1 diabetes, insulin, glucagon, polyhormonal cells

## Abstract

Type 1 diabetes (T1D) is related to the autoimmune destruction of β-cells, leading to their almost complete absence in patients with longstanding T1D. However, endogenous insulin secretion persists in such patients as evidenced by the measurement of plasma C-peptide. Recently, a low level of insulin has been found in non-β islet cells of patients with longstanding T1D, indicating that other islet cell types may contribute to persistent insulin secretion. The present study aimed to test the ability of various antibodies to detect insulin in insulin-deficient islets of T1D patients. Pancreatic autopsies from two children with recent-onset T1D, two adults with longstanding T1D, and three control subjects were examined using double immunofluorescent labeling with antibodies to insulin, glucagon and somatostatin. Immunoreactivity to insulin in glucagon+ cells of insulin-deficient islets was revealed using polyclonal antibodies and monoclonal antibodies simultaneously recognizing insulin and proinsulin. Along with this, immunoreactivity to insulin was observed in the majority of glucagon+ cells of insulin-containing islets of control subjects and children with recent-onset T1D. These results suggest that islet α-cells may contain insulin and/or other insulin-like proteins (proinsulin, C-peptide). Future studies are needed to evaluate the role of α-cells in insulin secretion and diabetes pathogenesis.

## 1. Introduction

Type 1 diabetes (T1D) is a severe pathology of the endocrine pancreas, which is related to the autoimmune destruction of insulin-secreting β-cells. Data from morphological studies indicate a significant reduction in β-cell mass in pancreatic autopsies and biopsies from patients with T1D [1,2,3,4,5,6,7,8,9,10,11,12,13,14]. Two morphologically distinct islet types have been described for the pancreas of persons with T1D—insulin-containing islets with preserved β-cells resembling normal islets, and insulin-deficient islets that lack β-cells [2,3,4,5]. An accurate estimation of the residual β-cell mass is difficult because insulin-containing islets are not randomly distributed within the pancreas but are instead located in several lobules, while the entire pancreas is often not available for analysis. Nevertheless, residual β-cells are detected in almost all cases of recent-onset T1D, and only in approximately 40% of cases of longstanding T1D [3,4,5,8,10,11,13,14]. The degree of β-cell loss varies between individuals and even between different lobes of the pancreas in the same individual [5,6,8,9]. In recent-onset T1D, the β-cell mass is reduced by approximately 60–90% [2,3,4,5,7,8]. Although some authors found no significant associations between β-cell mass and diabetes duration or age at onset [1,12], a meta-analysis of data from several morphological and biochemical studies indicated that, at the clinical onset of diabetes, younger children had a greater reduction of β-cell mass compared to teenagers and adults [15,16]. During the course of T1D, the β-cell mass continues to decrease, and, in patients with longstanding T1D, the reduction of the β-cell mass varies from 30% to 99.9% [3,4,5,9].

A gold standard for the estimation of residual β-cell function in diabetic patients is measurement of the plasma C-peptide level, including after glucagon stimulation or a mixed-meal test [17,18]. In agreement with histopathology findings, C-peptide is detected in the plasma of most patients with recent-onset diabetes [19,20], and higher C-peptide levels are observed in patients with an older age at diagnosis [20,21]. During the course of diabetes, the level of C-peptide decreases [20,22,23], and this decline in the C-peptide level is more rapid in patients with a younger age at diagnosis [23]. Also, many authors report that the proportion of patients with detectable levels of C-peptide decreases depending on the disease duration [19,21,24]. Nonetheless, a subset of patients with longstanding T1D has detectable C-peptide levels [20,21,24,25,26,27,28]. Current ultrasensitive approaches allow the detection of C-peptide in more than 60% of patients with a T1D duration of more than 30 years [25,26,27].

As generally accepted, the morphological source of this endogenous C-peptide in patients with longstanding T1D is preserved β-cells. Supporting this, a positive association between the C-peptide level and β-cell persistence in T1D has been shown in studies evaluating the C-peptide level and postmortem pancreas morphology in the same patients [6,9]. However, in a recent study of Lam and colleagues [29], a low level of insulin, which is undetectable using conventional methods, has been detected in the majority of insulin-deficient islets of patients with longstanding T1D using a long exposure technique. Cells with a low level of insulin often co-express α-cell phenotypic markers (glucagon, Arx), indicating that non-β islet cells may serve as a potential source of persistent insulin secretion in longstanding T1D.

In this study, the ability of various anti-insulin antibodies to detect insulin in insulin-deficient islets of T1D patients was tested. The cellular composition of insulin-deficient islets was compared with insulin-containing islets of T1D patients and control subjects.

## 2. Materials and Methods

This study was performed on pancreatic autopsy samples from the Collection of the Laboratory of Nervous System Development, Avtsyn Research Institute of Human Morphology, which were obtained from two children with recent-onset T1D, two adult patients with longstanding T1D, and three individuals not suffering from carbohydrate metabolism disorders (control group). T1D was diagnosed on the basis of a high blood glucose level and a young age at diagnosis. The duration of diabetes was measured as the time since T1D was first diagnosed. Available data concerning sex, age, disease duration, and the cause of death of diabetic and control subjects are given in Table 1. In two cases of recent-onset T1D, several fragments from different regions of the pancreas (head, body, and tail) were taken for analysis. In two patients with longstanding T1D and three control subjects, only one fragment from the body of the pancreas was available for analysis in each case. Tissue specimen collection and handling were performed in agreement with Russian legislation and the Declaration of Helsinki. Consent for use of the pancreatic tissue for research was obtained from a legal representative of each subject involved in the study. All protocols were approved by the local Ethics Committee of the Avtsyn Research Institute of Human Morphology (protocol No.33 (9), 7 February 2022).

Pancreatic samples were fixed in 10% buffered formalin (pH 7.0–7.4; BioVitrum, Saint Petersburg, Russia) within 8 h after death. Then, the samples were dehydrated in alcohols of increasing concentration and dioxane, embedded in paraffin, and, subsequently, a series of 4 μm thick sections were made for each sample. Sections were glued onto Superfrost slides (Thermo Fisher Scientific Inc., Fremont, CA, USA) and stored at 4 °C.

Indirect immunofluorescence (IF) using four different antibodies to insulin (Table 2) was applied to compare their ability to detect insulin immunoreactivity in cells of insulin-deficient islets. For IF labeling, sections of the pancreas were deparaffinized, rehydrated, and antigen retrieval by microwaving sections in 10 mM citric acid buffer, pH 6.0 for 10 min, and cooling for 20 min was performed. Then sections were incubated with a blocking solution consisting of 10% normal goat serum (Santa Cruz Biotechnology, Santa Cruz, CA, USA) in Tris-buffered saline plus 0.1% Tween 20 (TBST; Thermo Fisher Scientific Inc., Fremont, CA, USA) for 30 min at room temperature. Primary antibodies were diluted in 1% normal goat serum in TBST and incubated at 4 °C for 12 h. Optimal dilutions of the primary antibodies allowing detection of intense insulin staining along with minimal background staining were determined using a titrating assay on pancreatic sections from persons from the control group, and then applied on pancreatic sections from persons with T1D. Optimal dilutions of the primary antibodies are presented in Table 2. Secondary antibodies were diluted in TBST and incubated at room temperature for 2 h. The following secondary antibodies and dilutions were used: AlexaFluor^®^488 goat anti-mouse IgG (H+L) (1:200; Cat#A11029, RRID:AB_138404, Molecular Probes^®^; Thermo Fisher Scientific Inc., Eugene, OR, USA) and AlexaFluor^®^555 goat anti-rabbit IgG (H+L) (1:200; Cat#A21429, RRID:AB_141761, Molecular Probes^®^; Thermo Fisher Scientific Inc.).

To improve the detection of insulin immunoreactivity in cells of insulin-deficient islets, IF reactions with increased concentrations of the primary antibodies (lowest dilutions) (Table 2) and increased incubation time (from 12 h to 24 h) were performed on pancreatic sections from persons with T1D. Additionally, along with increased concentrations of the primary antibodies and 24 h incubation time, alternative secondary antibodies: IFluor™488 conjugated goat anti-mouse IgG (1:400, Cat#HA1125, Huabio, Hangzhou, China) and IFluor™488 conjugated goat anti-rabbit IgG (1:400, Cat#HA1121, Huabio, Hangzhou, China), which were incubated for 2 h at room temperature, were used.

To analyze colocalization of insulin with other islet cell hormones, double IF labeling was performed. As the distribution of the immunoreactivity to insulin differs among antibodies, the following combinations of primary antibodies were used: mouse monoclonal antibodies to insulin (1:16,000, Sigma) and rabbit polyclonal antibodies to glucagon (1:50, Thermo Fisher Scientific Inc.); mouse monoclonal antibodies to insulin (1:50, Thermo Fisher Scientific Inc.) and rabbit polyclonal antibodies to glucagon (1:50, Thermo Fisher Scientific Inc.); mouse monoclonal antibodies to insulin (1:16,000, Sigma) and rabbit polyclonal antibodies to somatostatin (1:50, Abcam); mouse monoclonal antibodies to insulin (1:50, Thermo Fisher Scientific Inc.) and rabbit polyclonal antibodies to somatostatin (1:50, Abcam); mouse monoclonal antibodies to glucagon (1:4000, Sigma) and rabbit polyclonal antibodies to somatostatin (1:50, Abcam). Primary antibodies diluted in 1% normal goat serum in TBST were applied on sections and incubated for 12 h at 4 °C. Secondary antibodies (1:200, AlexaFluor^®^488 goat anti-mouse IgG (H+L) and 1:200, AlexaFluor^®^555 goat anti-rabbit IgG (H+L)) were diluted in TBST and incubated at room temperature for 2 h.

Sections were covered using a mounting medium Fluoroshield™ with DAPI (Cat#F-6057, Sigma, St. Louis, MO, USA).

Negative control sections, in which primary antibodies were replaced with 1% normal goat serum in TBST, were included in every immunostaining procedure for each case. Additionally, negative control sections in which both primary and secondary antibodies were replaced with 1% normal goat serum in TBST were included to control for tissue autofluorescence.

Preparations were analyzed using an ADF U300 microscope equipped with a fluorescent block, digital microscopy camera Ultra09, and ADF Image capture software (version x64, 4.11.21522.20221011) (ADF Microscopes, Ningbo, Nanjing, China). All measurements were performed using the ADF Image capture software. The degree of β-cell loss was calculated on sections double-stained with antibodies to insulin (Thermo Fisher Scientific Inc.) and glucagon as the proportion of insulin-containing islets to the total number of glucagon-containing islets per section. For the analysis of the cellular composition, images of ten insulin-containing and ten insulin-deficient islets (for T1D patients) were captured from sections double-stained with antibodies to insulin (Sigma) and glucagon (Thermo Fisher Scientific Inc.); ten insulin-containing and ten insulin-deficient islets (for T1D patients) from sections double-stained with antibodies to insulin (Thermo Fisher Scientific Inc.) and somatostatin (Abcam); and ten insulin-containing and ten insulin-deficient islets (for T1D patients) from sections double-stained with antibodies to glucagon (Sigma) and somatostatin (Abcam). For each islet, the islet area, the total number of DAPI-positive nuclei, and the total number of a certain islet cell type were counted (for islets double-stained with antibodies to insulin and glucagon: insulin+, glucagon+, glucagon+/insulin+; for islets double-stained with antibodies to insulin and somatostatin: insulin+, somatostatin+, insulin+/somatostatin+; for islets double-stained with antibodies to glucagon and somatostatin: glucagon+, somatostatin+, glucagon+/somatostatin+). Then, the cell density (number of cells per µm^2^ of islet area) and the proportion of each cell type to the total cell number was calculated.

Statistical analysis was performed using the statistical software package Statistica 10 (Statsoft Inc., Tulsa, OK, USA). Data are expressed as medians (Me) and percentiles (P): 25P (q1–lower quartile) and 75P (q3–upper quartile). For multiple comparisons of the investigated parameters, the values of the islet area and cell density were integrated from three sets of data (islets double-stained for insulin and glucagon, islets double-stained for insulin and somatostatin, and islets double-stained for glucagon and somatostatin); the values of the proportion of somatostatin+ cells were integrated from two sets of data (islets double-stained for insulin and somatostatin and islets double-stained for glucagon and somatostatin); and the values of the proportion of insulin+ cells, the proportion of glucagon+/insulin+ cells, and the proportion of glucagon+ cells were used from one set of data (islets double-stained for insulin and glucagon). Correlations between different types of islet cells were analyzed separately for islets double-stained for insulin and glucagon, islets double-stained for insulin and somatostatin, and islets double-stained for glucagon and somatostatin. The nonparametric tests (multiple comparisons *p* values, Spearman rank R) were used because the values did not follow a Gaussian distribution and the numbers of observations in the examined groups were unequal. *p*-values below 0.05 were considered significant.

## 3. Results

### 3.1. Detection of Insulin in the T1D Pancreas

**Figure 1:**
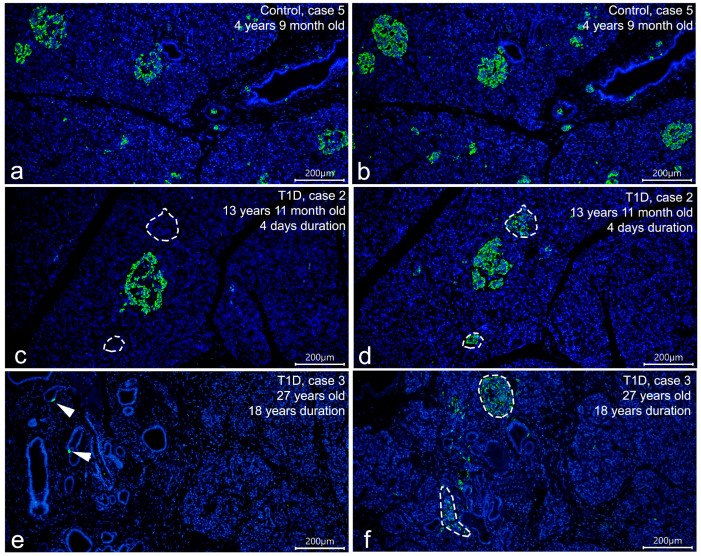
Distribution of the immunoreactivity to insulin (green) in control (**a**,**b**) and T1D (**c**–**f**) pancreas using different primary antibodies to insulin. IF staining with mouse monoclonal antibodies to insulin (Thermo Fisher Scientific Inc., MS-1379-P) (left panel; (**a**,**c**,**e**)) and mouse monoclonal antibodies to insulin (Sigma, I2018) (right panel; (**b**,**d**,**f**)); AlexaFluor^®^488 goat anti-mouse IgG (H+L) secondary antibodies; nuclei are stained by DAPI. (**a**,**b**) Adjacent sections of the pancreas of a child from the control group showing that both primary antibodies have similar distributions of immunoreactivity to insulin; insulin+ cells are seen in virtually all islets. (**c**,**d**) Adjacent sections of the pancreas of a child with recent-onset T1D illustrating differences in the distribution of immunoreactivity to insulin between antibodies. Mouse monoclonal antibodies produced by Thermo Fisher Scientific Inc. (**c**) show insulin immunoreactivity only in the preserved insulin-containing islets; in insulin-deficient islets (marked by dotted line), the reaction to insulin is negative. In the reaction with mouse monoclonal antibodies produced by Sigma (**d**), in addition to a positive reaction to insulin in insulin-containing islets, a less intense reaction is seen in the insulin-deficient islets. In longstanding T1D (**e**,**f**), immunoreactivity with mouse monoclonal antibodies produced by Thermo Fisher Scientific Inc. (**e**) is seen only in single cells (arrowheads) located within the ductal epithelium, while mouse monoclonal antibodies produced by Sigma (**f**) show additional insulin immunoreactivity in cells of insulin-deficient islets (marked by dotted line). Scale bar 200 µm.

To compare the ability of four different primary antibodies to insulin to detect insulin immunoreactivity in cells of insulin-deficient islets of T1D patients, IF protocols allowing the detection of intense insulin staining with minimal background staining were initially developed on pancreatic sections from persons from the control group. IF staining using optimal dilutions of the primary antibodies to insulin (listed in Table 2) and AlexaFluor^®^-conjugated secondary antibodies showed that all four primary antibodies had a similar distribution of immunoreactivity to insulin in the pancreas of persons from the control group; insulin+ β-cells were presented in virtually all pancreatic islets (Figure 1a,b).

**Figure 2:**
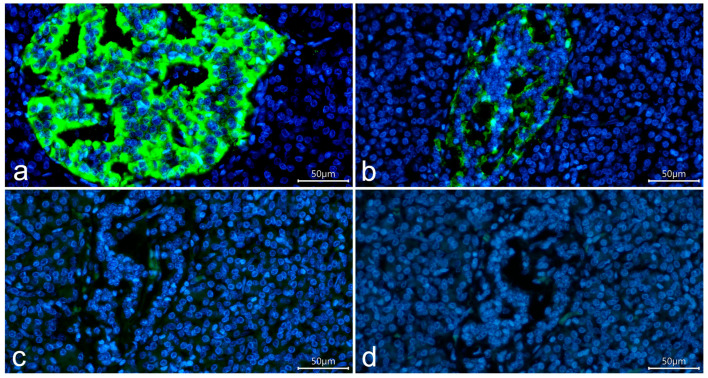
Pancreatic section of a child with recent-onset T1D (case 1, 3 years 9 month old, 6 days duration) stained with mouse monoclonal antibodies to insulin (Sigma, I2018) and AlexaFluor^®^488 goat anti-mouse IgG (H+L) secondary antibodies (green); nuclei are stained by DAPI. Intense reaction to insulin is seen in an insulin-containing islet (**a**), and less intense staining is seen in an insulin-deficient islet (**b**). (**c**,**d**) Sections adjacent to the section shown in (**b**) representing negative control sections in which primary antibodies were omitted (**c**) or both primary and secondary antibodies were omitted (**c**). No specific staining of islet cells is seen in negative control sections. Scale bar 50 µm.

Then, the same IF protocols were applied on pancreatic sections from persons with T1D, and significant differences in the distribution of the immunoreactivity to insulin between antibodies were observed. In the reactions with mouse monoclonal antibodies produced by Thermo Fisher Scientific Inc., rabbit polyclonal antibodies produced by Santa Cruz Biotechnology, and rabbit polyclonal antibodies produced by Abcam, insulin immunoreactivity was significantly reduced in the pancreas of patients with T1D (Figure 1c,e). In children with recent-onset T1D, there were only a few insulin-containing islets within the pancreas; the majority of islets were negative for insulin (insulin-deficient islets) (Figure 1c). The proportion of insulin-containing islets to the total number of islets in children with recent-onset T1D was estimated on sections double-stained with mouse monoclonal antibodies to insulin (Thermo Fisher Scientific Inc.) and glucagon. It was shown that the number of insulin-containing islets represented 11.96% in case 1 and 10.07% in case 2 (Table S1). In both cases of longstanding T1D, no insulin-containing islets were observed; in one of them (case 3), there were only single insulin+ cells located within the ductal epithelium (Figure 1e). In the reactions with mouse monoclonal antibodies produced by Sigma, along with a positive reaction to insulin in the preserved insulin-containing islets of children with recent-onset T1D and single insulin+ cells of patient with longstanding T1D, additional immunoreactivity to insulin was detected in all insulin-deficient islets (Figure 1d,f). The reaction to insulin in insulin-deficient islets was less intense than in insulin-containing islets (Figure 1d,f and Figure 2a,b).

In negative control sections in which primary antibodies were omitted (Figure 2c) or both primary and secondary antibodies were omitted (Figure 2d), no staining was observed in islet cells, indicating that the positive reaction to insulin in insulin-deficient islets was not due to nonspecific binding of secondary antibodies or tissue autofluorescence.

Thus, in IF protocols optimal for the detection of insulin in the control pancreas, positive reaction to insulin in cells of insulin-deficient islets was observed only with mouse monoclonal antibodies to insulin produced by Sigma, while the remaining three antibodies did not detect any insulin immunoreactivity in cells of insulin-deficient islets.

**Figure 3:**
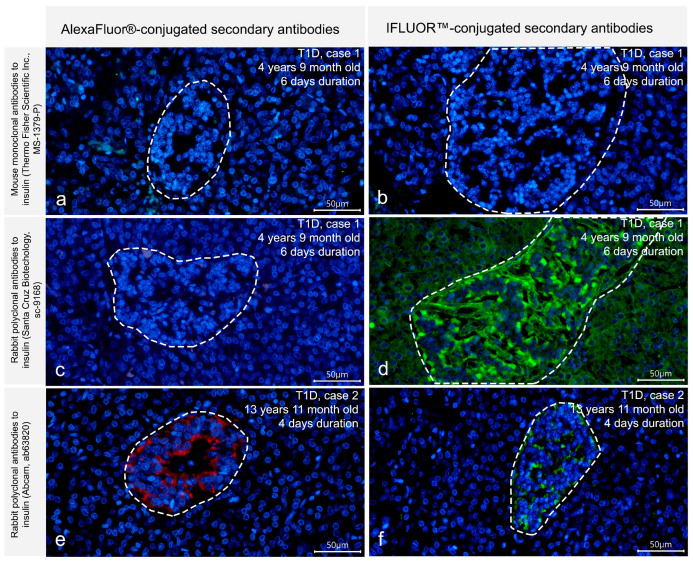
Detection of insulin immunoreactivity in insulin-deficient islets of children with recent onset T1D using different IF protocols. Left panel (**a**,**c**,**e**) demonstrates IF staining with increased concentrations of the primary antibodies (dilutions 1:50), increased incubation time (24 h) and AlexaFluor^®^-conjugated secondary antibodies; positive staining for insulin in insulin-deficient islets is seen with rabbit polyclonal antibodies produced by Abcam (ab63820) (**e**), but not with mouse monoclonal antibodies produced by Thermo Fisher Scientific Inc. (MS-1379-P) (**a**) or rabbit polyclonal antibodies produced by Santa Cruz Biotechnology (Sc-9168) (**c**). Right panel (**b**,**d**,**f**) demonstrates IF staining with increased concentrations of the primary antibodies (dilutions 1:50), increased incubation time (24 h) and IFluor™-conjugated secondary antibodies; positive staining for insulin in insulin-deficient islets is seen with rabbit polyclonal antibodies produced by Abcam (**f**) and rabbit polyclonal antibodies produced by Santa Cruz Biotechnology (**d**), while the reaction with mouse monoclonal antibodies produced by Thermo Fisher Scientific Inc. still remains negative (**b**). Insulin-deficient islets are marked by dotted lines. Nuclei are stained by DAPI. Scale bar 50 µm.

To test whether the observed differences in the distribution of immunoreactivity to insulin between antibodies were related to their sensitivity and a lower content of insulin in cells of insulin-deficient islets, IF staining with increased concentrations of primary antibodies (dilutions 1:50) and increased incubation time (24 h) was performed for mouse monoclonal antibodies produced by Thermo Fisher Scientific Inc., rabbit polyclonal antibodies produced by Abcam and rabbit polyclonal antibodies produced by Santa Cruz Biotechnology. Using these IF protocols, no insulin immunoreactivity in insulin-deficient islets was seen in the reactions with mouse monoclonal antibodies produced by Thermo Fisher Scientific Inc. (Figure 3a) or rabbit polyclonal antibodies produced by Santa Cruz Biotechnology (Figure 3c). However, positive staining for insulin in cells of insulin-deficient islets was observed in the reactions with rabbit polyclonal antibodies produced by Abcam (Figure 3e).

To further improve the detection of insulin in insulin-deficient islets, IF staining with IFluor™-conjugated secondary antibodies instead of AlexaFluor^®^-conjugated secondary antibodies along with an increased concentration of primary antibodies (dilutions 1:50) and increased incubation time (24 h) was performed. Using this approach, immunoreactivity to insulin in insulin-deficient islets was revealed with rabbit polyclonal antibodies produced by Abcam (Figure 3f), and, additionally, with rabbit polyclonal antibodies produced by Santa Cruz Biotechnology (Figure 3d). However, no positive staining for insulin in insulin-deficient islets was observed with mouse monoclonal antibodies produced by Thermo Fisher Scientific Inc. (Figure 3b).

In general, the obtained results indicate the presence of insulin immunoreactivity in cells of insulin-deficient islets of T1D patients. The ability to detect this insulin immunoreactivity differs among various primary antibodies. Among the four antibodies used in this study, two rabbit polyclonal antibodies (produced by Abcam and Santa Cruz Biotechnology) showed a positive reaction to insulin in cells of insulin-deficient islets only when increased concentrations, increased incubation time and more sensitive secondary antibodies were applied, suggesting a lower content of antigen in cells of insulin-deficient islets. At the same time, mouse monoclonal antibodies produced by Sigma were suitable for the detection of insulin immunoreactivity in insulin-deficient islets in IF protocols optimal for the control pancreas, indicating that not only sensitivity but also specificity of the primary antibodies may influence the detection of insulin immunoreactivity in cells of insulin-deficient islets.

### 3.2. Co-Localization of Insulin with Glucagon and Somatostatin

**Figure 4:**
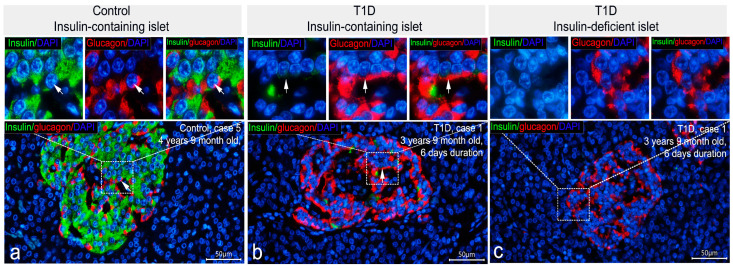
Double IF staining with mouse monoclonal antibodies to insulin (Thermo Fisher Scientific Inc., MS-1379-P) (green) and rabbit polyclonal antibodies to glucagon (red); nuclei are stained by DAPI. In insulin-containing islets of control (**a**) and T1D (**b**) persons, the majority of cells contain either insulin or glucagon; co-localization of both hormones is seen only in rare cells (arrows). Insulin-deficient islets of T1D patients predominantly composed of glucagon+ cells in which the reaction to insulin is negative (**c**). Scale bar 50 µm.

To determine islet cell types demonstrating insulin immunoreactivity in insulin-deficient islets, double IF staining was performed. Due to differences in the distribution of immunoreactivity to insulin between primary antibodies, the co-localization of insulin and glucagon was compared using the following combinations: mouse monoclonal antibodies to insulin, Thermo Fisher Scientific Inc. (negative reaction to insulin in insulin-deficient islets) + rabbit polyclonal antibodies to glucagon; and mouse monoclonal antibodies to insulin, Sigma (positive reaction to insulin in insulin-deficient islets) + rabbit polyclonal antibodies to glucagon. Analysis of sections stained with the first combination of antibodies demonstrated that in insulin-containing islets of control subjects and patients with recent-onset T1D, the majority of cells contained either insulin or glucagon (Figure 4a,b). Co-localization of insulin and glucagon was found only in a small number of cells (0–3 per islet) (Figure 4a,b). As expected, insulin-deficient islets of patients with T1D were predominantly composed of glucagon+ cells in which the reaction to insulin was negative (Figure 4c).

**Figure 5:**
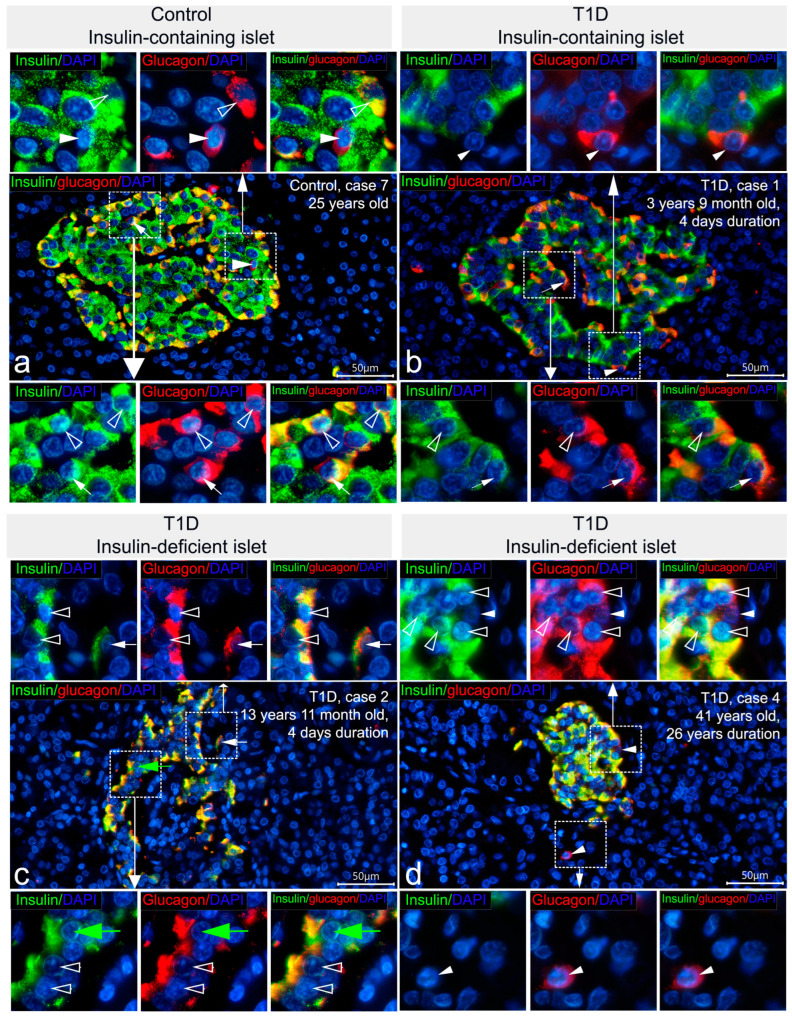
Double IF staining with mouse monoclonal antibodies to insulin (Sigma, I2018) (green) and rabbit polyclonal antibodies to glucagon (red); nuclei are stained by DAPI. The majority of glucagon+ cells in insulin-containing islets of control (**a**) and T1D (**b**) persons, as well as glucagon+ cells in insulin-deficient islets of T1D persons (**c**,**d**), show strong immunoreactivity to insulin (some cells with co-localization of glucagon and insulin are marked by empty arrowheads). There are very rare cells stained for only glucagon (white arrowheads). White arrows indicate cells in which insulin and glucagon immunoreactivity are distributed in different areas of the cytoplasm. Green arrows indicate cells located in the insulin-deficient islets and positive for only insulin. Scale bar 50 µm.

Double IF staining with mouse monoclonal antibodies to insulin produced by Sigma and glucagon revealed numerous cells double-positive for insulin and glucagon both in control and T1D pancreatic samples (Figure 5). In insulin-deficient islets of T1D patients, the majority of glucagon+ cells showed immunoreactivity to insulin (glucagon+/insulin+ cells), and cells positive for glucagon only and for insulin only were very rare (Figure 5c,d). Along with this, in insulin-containing islets of control subjects and patients with recent-onset T1D, almost all glucagon-positive cells were simultaneously positive for insulin (Figure 5a,b); there were also β-cells positive for insulin only and very rare cells positive for glucagon only (Figure 5a,b).

The intracellular distribution of immunoreactivity to insulin and glucagon varied from cell to cell. In insulin-containing islets of control subjects and patients with T1D, the majority of glucagon+/insulin+ cells had similar localization of immunopositive granules in the cytoplasm (Figure 5a,b and Figure S1a–h). In some cells, insulin- and glucagon-immunoreactivity were distributed in different areas of the cytoplasm (Figure 5a,b and Figure S1a–d).

High variability in the content of granules in glucagon+/insulin+ cells was also observed. There were cells with a large amount of insulin+ and a large amount of glucagon+ granules; cells with few insulin+ and few glucagon+ granules; and cells with a predominance of either insulin+ or glucagon+ granules (Figure S1a–h). In insulin-deficient islets of T1D patients, almost all double-positive cells had a similar distribution of insulin+ and glucagon+ granules in the cytoplasm (Figure 5c,d and Figure S1i–l). In some cells, the prevalence of insulin+ or glucagon+ granules, as seen by the intensity of the insulin and glucagon staining and localization of the insulin+ and glucagon+ granules in different areas of the cytoplasm, was apparent (Figure 5c and Figure S1i–l).

**Figure 6:**
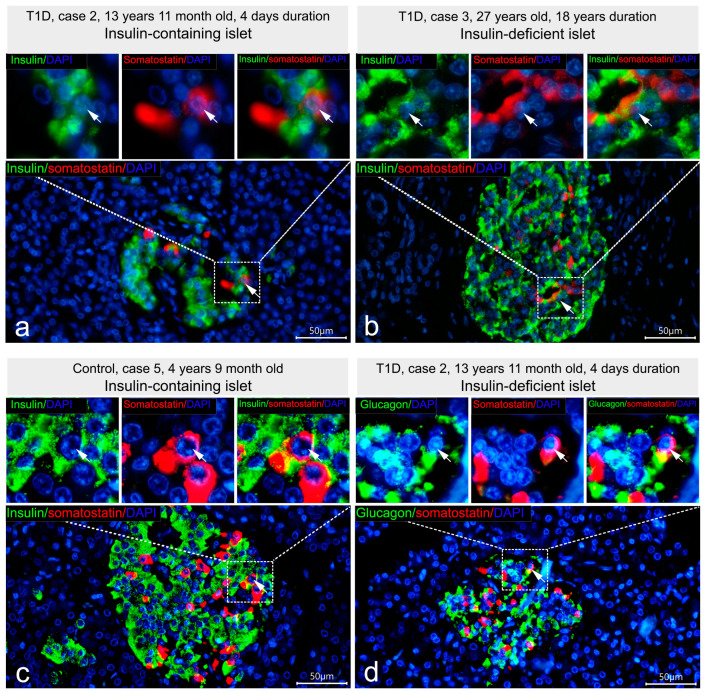
Co-localization of insulin and somatostatin in insulin-containing islets of children with recent-onset T1D (**a**), insulin-containing islets of control subjects (**c**), and insulin-deficient islets of T1D patients (**b**,**d**). (**a**,**b**) Double IF staining with mouse monoclonal antibodies to insulin (Sigma, I2018) (green) and rabbit polyclonal antibodies to somatostatin (red); (**c**) double IF staining with mouse monoclonal antibodies to insulin (Thermo Fisher Scientific Inc., MS-1379-P) (green) and rabbit polyclonal antibodies to somatostatin (red); (**d**) double IF staining with mouse monoclonal antibodies to glucagon (Sigma, G2654) (green) and rabbit polyclonal antibodies to somatostatin (red). Nuclei are stained by DAPI. White arrows indicate rare cells with co-localization of somatostatin with either insulin or glucagon. Scale bar 50 µm.

Analysis of the co-localization of insulin and somatostatin was initially performed using double IF staining with mouse monoclonal antibodies to insulin produced by Sigma and rabbit polyclonal antibodies to somatostatin. It was shown that in islets of control subjects and in all types of islets of patients with T1D the majority of somatostatin-positive cells did not contain insulin (Figure 6a,b).

Co-localization of insulin and somatostatin was observed only in a few cells, which were found in all types of islets in both control and T1D groups (Figure 6a,b). Within such cells, insulin- and somatostatin-positive granules were frequently distributed in different parts of the cytoplasm. To distinguish whether somatostatin co-localized with insulin or also with glucagon, double IF staining using mouse monoclonal antibodies to insulin (Thermo Fisher Scientific Inc.) (negative staining for insulin in insulin-deficient islets) and rabbit polyclonal antibodies to somatostatin; and mouse monoclonal to glucagon (Sigma) and rabbit polyclonal antibodies to somatostatin was performed. It was revealed that in insulin-containing islets of control subjects and children with recent-onset T1D, somatostatin co-localized with both insulin Figure 6c) and glucagon, while in insulin-deficient islets, only cells with co-localization of glucagon and somatostatin were observed (Figure 6d).

Thus, the additional insulin immunoreactivity, which is detectable using mouse monoclonal antibodies to insulin produced by Sigma, was mostly co-localized with glucagon, but not with somatostatin. Moreover, when insulin immunoreactivity was detectable in glucagon+ cells of insulin deficient islets, it simultaneously appeared in glucagon+ cells of insulin-containing islets of children with recent-onset T1D and control subjects. Therefore, glucagon+ cells of insulin-deficient islets showing positive staining for insulin may represent α-cells similar to that of insulin-containing islets.

### 3.3. Cellular Composition of Insulin-Containing and Insulin-Deficient Islets

**Figure 7:**
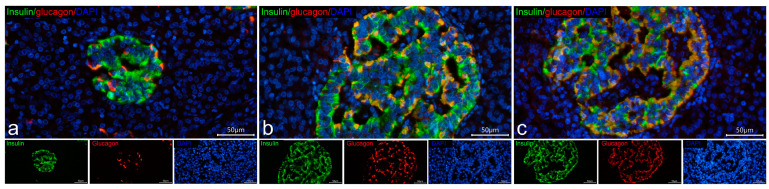
Cellular composition of insulin-containing islets in a recent-onset T1D (case 1, 3 years 9 months old, 6 days duration). Double IF staining with mouse monoclonal antibodies to insulin (Sigma, I2018) (green) and rabbit polyclonal antibodies to glucagon (red); nuclei are stained by DAPI. The proportion of different types of islet cells varies—from islets with a predominance of insulin+ cells (**a**) to islets with approximately equal proportions of insulin+ and glucagon+/insulin+ cells (**b**), and islets with a predominance of glucagon+/insulin+ cells (**c**). Scale bar 50 µm.

The cellular composition of insulin-containing and insulin-deficient islets was compared to evaluate the relationships between these two islet types and to estimate the role of glucagon+ showing insulin immunoreactivity in the formation of insulin-deficient islets. Insulin-deficient islets of patients with T1D were composed predominantly of glucagon+ cells showing positive staining for insulin with mouse monoclonal antibodies produced by Sigma (glucagon+/insulin+ cells), less frequent somatostatin+ cells, and very rare cells positive for only insulin or only glucagon (Figure 5c,d and Figure 6b). The main difference between insulin-containing islets and insulin-deficient was the presence of monohormonal β-cells. In control subjects, insulin-containing islets were composed mostly of fully granulated insulin+ β-cells with a smaller proportion glucagon+/insulin+ cells (Figure 5a); somatostatin+ cells were less frequent, and cells positive for only glucagon and somatostatin+/insulin+ cells were found very rarely. The same islet cell types were presented in insulin-containing islets of children with recent-onset T1D (Figure 5b, Figure 6a and Figure 7). However, in insulin-containing islets of children with recent-onset T1D, a large proportion of insulin+ β-cells demonstrated various degrees of degranulation (Figure 7), and these islets had highly variable proportions of insulin+ and glucagon+/insulin+ cells—from islets with a predominance of insulin+ cells (Figure 7a) to islets with approximately equal proportions of insulin+ and glucagon+/insulin+ cells (Figure 7b), and islets with a predominance of glucagon+/insulin+ (Figure 7c).

Therefore, within the islet, a decrease in the proportion of insulin+ β-cells could be accompanied by a corresponding increase in the proportion of glucagon+/insulin+ cells, and islets with a decreased proportion of insulin+ cells and increased proportion of glucagon+/insulin+ cells and other islet cell types may represent transient forms between insulin-containing and insulin-deficient islets.

**Figure 8:**
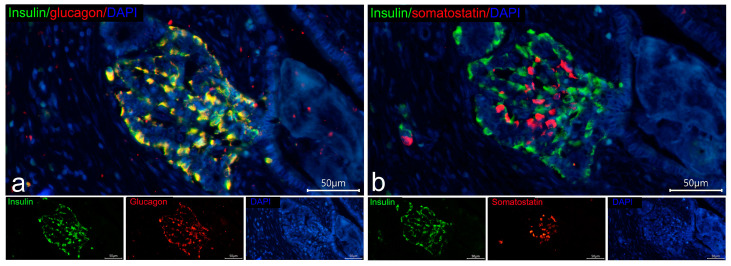
Adjacent sections of the pancreas of a patient with longstanding T1D (case 3, 27 years old, 18 years duration) demonstrating insulin-deficient islets associated with ductal epithelium (islet–duct complex). Double IF staining with mouse monoclonal antibodies to insulin (Sigma, I2018) and rabbit polyclonal antibodies to glucagon (red) (**a**); mouse monoclonal antibodies to insulin (Sigma, I2018) (green) and rabbit polyclonal antibodies to somatostatin (red) (**b**); nuclei are stained by DAPI. Scale bar 50 µm.

As pancreatic islets are known to be formed from the ductal epithelium, interactions between insulin-deficient islets and pancreatic ducts (islet–duct complexes) were investigated. The majority of insulin-deficient islets were located within the acinar parenchyma or in the interlobular connective tissue. In one case of longstanding T1D, insulin-deficient islets associated with the ductal epithelium were found (Figure 8), indicating that islets lacking fully granulated β-cells may arise from the ductal epithelium.

### 3.4. Quantitative Morphometric Analysis of Cellular Composition of Insulin-Containing and Insulin-Deficient Islets

To confirm the qualitative observations, quantitative morphometric per islet analysis was performed. The relationships between insulin+ and glucagon+/insulin+ cells were analyzed on sections double-stained with mouse monoclonal antibodies to insulin (Sigma) and rabbit polyclonal antibodies to glucagon (Thermo Fisher Scientific Inc.). Since it was impossible to distinguish true β-cells from glucagon+/insulin+ cells using mouse monoclonal antibodies to insulin produced by Sigma, the relationships between insulin+ cells and somatostatin+ cells were analyzed on sections double-stained with mouse monoclonal antibodies to insulin (Thermo Fisher Scientific Inc.) and somatostatin (Abcam), and the relationships between glucagon+ cells and somatostatin+ cells were determined on sections double-stained with mouse monoclonal antibodies to glucagon (Sigma) and somatostatin (Abcam). Thus, three sets of data were obtained. All initial measurements and subsequent calculations for each islet are presented in Tables S2–S4; values of median and interquartile ranges for the investigated morphometric parameters are presented in Table 3.

**Figure 9:**
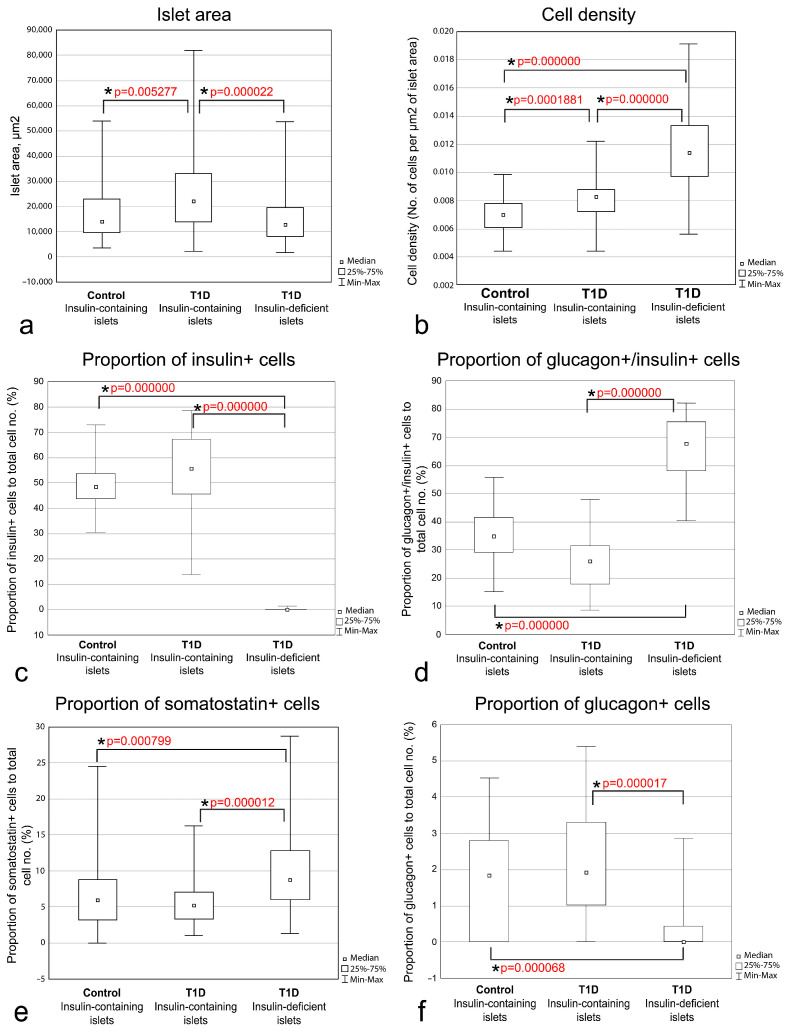
Boxplots and multiple comparison *p*-values for islet area (**a**), cell density (**b**), proportion of insulin+ cells (**c**), proportion of glucagon+/insulin+ cells (**d**), proportion of somatostatin+ cells (**e**), proportion of glucagon+ cells (**f**) in insulin-containing islets of control subjects, insulin-containing islets of T1D patients, and insulin-deficient islets. Significant differences between groups are marked by square brackets and asterisks.

Comparisons of the morphometric parameters in various types of islets showed that the size of insulin-deficient islets resembled the size of insulin-containing islets of control subjects (Table 3, Figure 9a). In children with recent-onset T1D, the size of insulin-containing islets was increased (Table 3, Figure 9a). The cell density was increased in islets of persons with T1D compared to control subjects, and, in persons with T1D, cells were distributed more densely within insulin-deficient islets than in insulin-containing islets (Table 3, Figure 9b), indicating that an increase of cell density may occur during the formation of insulin-deficient islets.

The proportion of insulin+ cells was not significantly different between insulin-containing islets of control subjects and children with recent-onset T1D (48 and 56% respectively), but was reduced in insulin-deficient islets (0%) (Table 3, Figure 9c). At the same time, a significant increase in the proportion of glucagon+/insulin+ cells in insulin-deficient islets was observed; 68% in insulin-deficient islets vs. 35% and 26% in insulin-containing islets of control subjects and children with recent-onset T1D, respectively (Table 3, Figure 9d). The proportion of somatostatin+ cells was also increased in insulin-deficient islets of persons with T1D (9%) compared to insulin-containing islets of control subjects (6%) and persons with T1D (5%) (Table 3, Figure 9e). These findings confirm that the formation of insulin-deficient islets could be accompanied not only by a decrease in the proportion of insulin+ cells, but also by an increase in the proportion of glucagon+/insulin+ cells and somatostatin+ cells.

It was also found that the proportion of cells positive for only glucagon (glucagon+ cells) was decreased in insulin-deficient islets comparing to insulin-containing islets of control subjects and children with recent-onset T1D (Table 3, Figure 9f), but these differences were possibly related to the small number of glucagon+ cells and higher content of glucagon+/insulin+ cells in insulin-deficient islets.

The proportion of cells double-positive for insulin and somatostatin (insulin+/somatostatin+) and double-positive for glucagon and somatostatin (glucagon+/somatostatin+) was very low (less than 1% in all types of islets) (Table 3). The proportion of insulin+/somatostatin+ cells was 0%, 0.4% and 0% in insulin-containing islets of control subjects, insulin-containing islets of persons with T1D, and insulin-deficient islets, respectively, and was significantly lower in insulin-deficient islets than in insulin-containing islets of persons with T1D (Table 3, Figure S2a), which was due to the absence of true β-cells in insulin-deficient islets. The proportion of glucagon+/somatostatin+ cells in insulin-deficient islets of persons with T1D (0.9%) was significantly higher than in insulin-containing islets of control subjects (0%), but not different from insulin-containing islets of persons with T1D (0.4%) (Table 3, Figure S2b).

**Figure 10:**
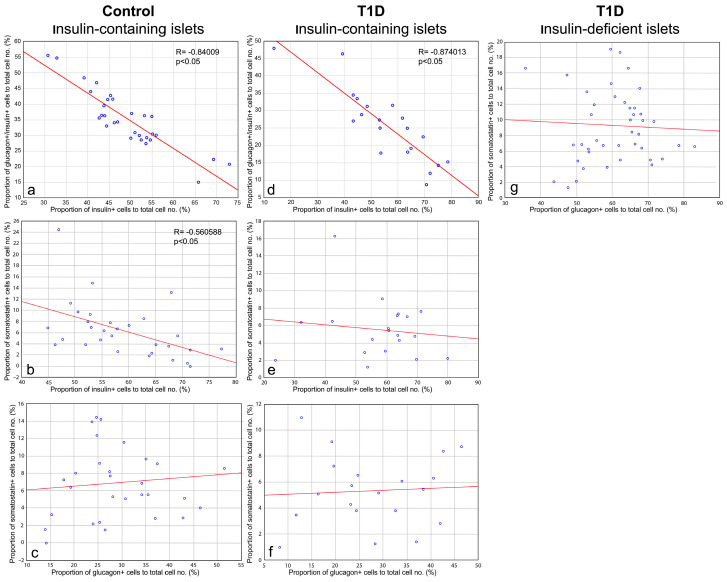
Scatterplots and Spearman rank correlations of the proportion of insulin+ cells and glucagon+/insulin+ cells (**a**,**d**), the proportion of insulin+ cells and somatostatin+ cells (**b**,**e**), and the proportion of glucagon+ cells and somatostatin+ cells in insulin-containing islets of control subjects (**a**–**c**), insulin-containing islets of children with recent-onset T1D (**d**–**f**), and insulin-deficient islets (**g**).

To further evaluate the relationships among various types of islet cells, correlation analysis was performed. A high negative correlation between the proportion of insulin+ cells and glucagon+/insulin+ cells was observed in insulin-containing islets of both control subjects (Figure 10a) and children with recent-onset T1D (Figure 10b), confirming that within the islet, a decrease in the proportion of insulin+ cells may be accompanied by an increase in the proportion of glucagon+/insulin+ cells and vice versa. There was also a medium negative correlation between the proportion of insulin+ cells and somatostatin+ cells in insulin-containing islets of control subjects (Figure 10c), but no correlation between these parameters was observed in insulin-containing islets of persons with T1D (Figure 10d). In all types of islets, no correlations were observed between the proportion of glucagon+ and somatostatin+ cells (Figure 10e–g).

It was also found that the proportion of insulin+/somatostatin+ cells correlated with the proportion of somatostatin+ cells in insulin-containing islets of children with T1D (Figure S2h), and the proportion of glucagon+/somatostatin+ cells correlated with the proportion of glucagon+ cells in insulin-containing islets of children with T1D (Figure S2e) and with the proportion of somatostatin+ cells in insulin-containing islets of children with T1D (Figure S2j) and in insulin-deficient islets (Figure S2l). However, these correlations were not reproduced in different types of islets (Figure S2). Thus, no strong relationships between the proportion of insulin+/somatostatin+ cells and glucagon+/somatostatin+ cells with other types of islet cells were found.

Based on the quantitatively confirmed relationship between the proportion of insulin+ cells and glucagon+/insulin+ cells in the islets, and on the presence of transient forms between insulin-containing and insulin-deficient islets in the pancreas of children with recent-onset T1D, it can be assumed that insulin-deficient islets are formed from the insulin-containing islets as a result of the loss of insulin+ cells and an increase in the proportion of glucagon+/insulin+ cells. Since some increase in the proportion of somatostatin+ cells was observed in insulin-deficient islets, and a medium negative correlation between the proportion of insulin+ cells and somatostatin+ cell was found in insulin-containing islets of control subjects, it is also possible that an increase in the proportion of somatostatin+ cells may occur during the formation of insulin-deficient islets.

## 4. Discussion

Immunohistochemical staining for insulin is widely used to identify β-cells in the pancreas and estimate their mass. However, in several studies, the problem of underestimation of β-cell mass in the diabetic pancreas by using this conventional approach was noted. Both in animal models and humans with type 2 diabetes, a significant decrease in insulin staining observed at the light microscope level was shown to be due to significant β-cell degranulation rather than β-cell death as seen at the electron microscope level [30,31,32]. These poorly granulated β-cells can be identified using special approaches. For example, cells with low insulin content have been identified in the islets of newly diabetic NOD mice by using two different secondary antibodies [33], and in humans with longstanding T1D using a long exposure technique [29].

The findings of the present study are in agreement with previous data and indicate the presence of insulin immunoreactivity in cells of insulin-deficient islets of T1D patients. Among four applied primary antibodies, two (a rabbit polyclonal produced by Abcam and one from Santa Cruz Biotechnology) allowed the detection of insulin immunoreactivity only with more sensitive IF protocols, while mouse monoclonal antibodies produced by Sigma showed positive insulin staining in cells of insulin-deficient islets in IF protocols optimal for the control pancreas. These differences in the immunoreactivity to insulin between antibodies were likely caused by differences in their sensitivity and specificity. Indeed, immunoreactivity to insulin in insulin-deficient islets was less intense than in insulin-containing islets, suggesting that cells of insulin-deficient islets have a lower content of antigen, which can be poorly detectable for some antibodies. The specificity of various antibodies, i.e., the ability of antibodies to interact with different epitopes of antigen, may also influence the distribution of immunoreactivity to insulin. 

It is well known that insulin biosynthesis occurs through a cleavage of the prohormone (proinsulin) consisting of the A-chain and B-chain of insulin connected by the C-peptide (connecting peptide) [34]. Polyclonal antibodies raised against insulin often cross-react with the prohormone [35]. Different clones of antibodies to insulin recognize various epitopes on the A-chain and B-chain [35,36,37], but some epitopes are not recognizable when, for example, they are connected to C-peptide in proinsulin [37]. On the other hand, some clones of antibodies to insulin are able to recognize proinsulin [35]. In particular, mouse monoclonal antibodies to insulin (Clone K36aC10) used in the present study have reported cross-reactivity with proinsulin [35]. In cells of insulin-deficient islets immunoreactivity to insulin was detected using polyclonal antibodies and monoclonal antibodies simultaneously recognizing hormone and prohormone. Therefore, it can be assumed that cells of insulin-deficient islets contain a low level of insulin along with proinsulin, or proinsulin alone, which is better detected by antibodies simultaneously recognizing both hormone and prohormone. Data of Lam and colleagues [29] showing immunoreactivity to insulin, A-chain, B-chain, and C-peptide in cells of insulin-deficient islets also confirmed that these cells may contain both hormone and prohormone.

As was revealed, additional insulin immunoreactivity, which is detectable using antibodies produced by Sigma, was co-localized with glucagon in cells of insulin-deficient islets. Along with this, positive staining for insulin appeared in the majority of glucagon+ cells of insulin-containing islets of control subjects and children with recent-onset T1D. Despite the majority of glucagon+ cells being stained for insulin using antibodies produced by Sigma, and the immunoreactivity to both insulin and glucagon was often distributed in the same areas of the cytoplasm, in all investigated pancreatic samples, and in all types of islets, rare cells positive for only glucagon were found, as well as cells in which immunoreactivity to insulin and glucagon was seen in different granules located in different areas of the cytoplasm. Thus, it seems unlikely that the staining for insulin in glucagon+ cells represents nonspecific staining of glucagon. Previously, the coexpression of insulin and glucagon was observed only in a small proportion of islet cells in nondiabetic subjects, and some increase in the number of such cells was detected in persons with type 2 diabetes [38,39,40]. Similar results showing a small proportion of insulin+/glucagon+ islet cells in normoglycemic conditions with an increase in their proportion in diabetes were obtained in rodents [30]. Lam and colleagues also did not find a low level of insulin in glucagon+ cells of control islets using a long exposure technique [29]. However, only rare control islets that lacked β-cells and mainly comprised α-cells were analyzed in this study, since the intense insulin staining of β-cells in control islets did not allow for excluding any low-level insulin within typical control islets when a long exposure was used [29]. It can be suggested that the number of identified glucagon+/insulin+ cells also depends on the sensitivity and specificity of antibodies to insulin. The application of more sensitive antibodies allowed detection of insulin in insulin-deficient islets without additional techniques, make it easier to detect insulin immunoreactivity in glucagon+ cells of insulin-containing islets. In studies devoted to single cell RNA sequencing of isolated pancreatic islets, including those from donors with T1D, cell clusters expressing both INS and GCG transcripts have been reported, but were excluded from the analysis as possible doublets or low-quality cells [41,42,43]. Nonetheless, as seen from data obtained in these studies, cells annotated as α-cells (clusters expressing GCG and other α-cell specific genes at a high level) also express INS transcripts at a lower level, and vice versa, cells annotated as β-cells (clusters expressing INS and other β-cell specific genes at a high level) also express GCG transcripts at a lower level [41,42,43]. Although the presence of the transcripts of INS in α-cells itself does not guarantee the presence of a protein product, this may serve as indirect evidence for the presence of proinsulin and insulin in α-cells. Thus, the majority of glucagon+ α-cells may contain insulin or other insulin-like proteins (proinsulin, C-peptide), and the ability to detect insulin immunoreactivity in glucagon+ cells depends on the applied antibodies and sensitivity of the approach. This fact should be taken into account in studies devoted to the estimation of the β-cells mass, because the number insulin+ cells may be overestimated when more sensitive antibodies and approaches are applied.

The presence of insulin in glucagon+ cells reflects the high phenotypic plasticity of pancreatic islet cells. In patients with longstanding T1D, cells with a low level of insulin co-express markers of β- (Nkx6.1, Pdx1, PC1/3, and GLUT1) and α- (glucagon, ARX, GLP-1, and GC) cells sharing a mixed β/α phenotype; some SS-, PP-, and ghrelin cells also express a low level of insulin [29]. Expression of the other β-cell specific marker, Pdx1, was also seen in glucagon-positive cells of insulin-deficient islets in pancreatic biopsies from patients with T1D [44]. Similar alterations in the islet cell phenotype occur in type 2 diabetes, in which an increase in the proportion of islet cells co-expressing insulin, glucagon and Nkx6.1, and glucagon with Nkx6.1, was found [39]. Some authors propose that non-β islet cells might alter their function to compensate for insulin deficiency in T1D [29]. On the other hand, data from experimental studies suggest that dedifferentiation of β-cells into immature phenotypes and transdifferentiation into other types of islet cells occur in diabetic conditions, and this loss of β-cell identity contributes to the reduction in β-cell mass [30,33,45]. As shown in mouse models of diabetes and rats after near total pancreatectomy, hyperglycemia leads to significant β-cell degranulation, a reduction in insulin content, decreased expression of mature β-cell markers (Pdx1, Nkx6.1, and MafA), and elevated expression of progenitor cell markers, such as Ngn3, Nanog, and L-Myc [30,45,46,47]. Furthermore, some dedifferentiated β-cells co-express insulin and glucagon or glucagon alone both in rodents [30] and in humans with type 2 diabetes [39,40], suggesting the transdifferentiation of β-cells into α-cells. This was also confirmed by studies with a deletion of key β-cell transcriptional factors demonstrating the reprogramming of β-cells into other islet cell types, such as into α-cells when Pdx1 is deleted [48], and into α-, δ-, and PP-cells when Nkx2.2 is deleted [49]. Thus, glucagon+ cells with a low level of insulin in insulin-deficient islets may represent α-cells expressing insulin or dedifferentiated/transdifferentiated β-cells. Since similar insulin immunoreactivity was observed in α-cells of insulin-containing islets of control subjects, at least some of the glucagon+/insulin+ cells in insulin-deficient islets may represent α-cells. At the same time, since the study by Gepts [5] who noted that small cells in insulin-deficient islets could not be distinguished as α-cells or undifferentiated cells, a number of pieces of evidence suggesting differences between cells of insulin-deficient islets and true α-cells were obtained. In particular, cells of insulin-deficient islets express β-cell specific markers (Pdx1 and Nkx6.1) while α-cells do not [29,44].

As shown in animal models of diabetes, the loss of β-cell identity (dedifferentiation into immature phenotypes and transdifferentiation into other islet cell types) resulted in decreased insulin staining in islets of diabetic animals [30,33,45,47]. This decrease in the number of insulin+ β-cells was accompanied by an equivalent increase in the number of glucagon+ cells [30,45]. In the present study, similar morphological alterations, such as degranulation of β-cells, and the appearance of insulin-containing islets with a decreased proportion of insulin+ cells and an increased proportion of glucagon+/insulin+ cells were observed in the pancreas of children with recent-onset T1D. Moreover, in the insulin-containing islets of both control subjects and children with recent-onset T1D, a negative correlation between the proportion of insulin+ cells and glucagon+/insulin+ cells was observed. In insulin-containing islets of control subjects, there was a weaker correlation between the proportion of insulin+ and somatostatin+ cells. These facts confirm that a decrease in the relative number of insulin+ cells could be accompanied by an increase in the relative number of glucagon+/insulin+ cells and probably other types of islet cells and vice versa. It was also shown, that in insulin-deficient islets of T1D patients the proportions of glucagon+/insulin+ cells and somatostatin+ cells were significantly higher than in insulin-containing islets. It can be therefore assumed that islets with a reduced proportion of insulin+ cells and an increased proportion of glucagon+/insulin+ cells and other types of islet cells may represent transient forms between normal insulin-containing islets and insulin-deficient islets. In this case, insulin-deficient islets may form as a result of degranulation, dedifferentiation of β-cells, and subsequent activation of the α-cell phenotypic program as was shown in hyperglycemic conditions in animal models of diabetes [30,33]. Importantly, in animal models of diabetes, pharmacological intervention at initial stages of the disease leads to the restoration of β-cell mass [30,33]. This restoration occurs through regranulation of dedifferentiated or transdifferentiated cells rather than through proliferation or neogenesis of β-cells [30,33]. Therefore, analysis of the phenotypes of cells present in insulin-deficient islets of persons with T1D is of great importance for researching possible sources for the restoration of the β-cell mass in patients with diabetes and developing therapeutic approaches for the treatment of the disease.

At the same time, in one case of longstanding T1D, insulin-deficient islets closely associated with pancreatic ductal epithelium (islet-duct complexes) were found. This suggests that islet neogenesis persists even in patients with a long disease duration, but these newly formed islets completely lack fully granulated monohormonal β-cells. Of course, the exact causes underlying the absence of β-cell cannot be determined in studies of human pancreatic autopsies, but it can be suggested that this may be due to impaired β-cell differentiation and maturation. The presence of bihormonal (insulin+/glucagon+) cells in the developing human pancreas has been shown in a number of studies using immunohistochemistry [50,51], immunocytochemistry [52], single-cell RNA sequencing, and spatial transcriptomics [53]. It was also demonstrated that some glucagon+ cells express β-cell specific transcriptional factors during pancreas development [54], and the proportion of insulin+/glucagon+ cells decreases as development proceeds, indicating that such cells may represent certain stages of differentiation of islet cells (including β-cells) [51,52,53]. Therefore, it is possible that at least some of the glucagon+/insulin+ cells in insulin-deficient islets may be differentiated β-cells, which cannot acquire a mature β-cell phenotype. This may occur due to the impact of diabetic conditions (chronic hyperglycemia, lipotoxicity, hypoxia, etc.) on the expression of key β-cell transcriptional factors, as it was, for example, shown in primates given a high fat/high sugar diet, in which decreased expression of FOXO1, NKX6.1, NKX2.2 and IPF1 (Pdx1) was observed, resulting in a switch from the β- to the α-cell phenotype [55].

The presence of a polyhormonal stage in the differentiation of endocrine cells was also confirmed by the data from in vitro studies devoted to the derivation of human insulin producing β-cells. Many protocols of differentiation of human embryonic and induced pluripotent stem cells promote generation of insulin-containing cells that are polyhormonal (co-express insulin and glucagon) and non-functional, which is probably caused by their precocious endocrine differentiation [56,57,58,59]. Of note, recapitulation of signaling pathways regulating normal pancreas development allow for the generation of monohormonal functionally active β-cells. In particular, the induction of endocrine differentiation in PDX1+ progenitors resulted in the appearance of multiple polyhormonal cells, while the induction of endocrine differentiation after the activation of NKX6.1 in PDX1+ progenitors resulted in the differentiation of PDX1+/NKX6.1+ progenitors into monohormonal functionally active β-cells [58,59].

At present, impaired maturation of β-cells, which includes alterations in insulin biosynthesis and its accumulation in secretory granules, is considered one of the possible causes of insulin deficiency in both type 1 and type 2 diabetes. According to data from biochemical studies, many patients with T1D have detectable serum proinsulin even in the absence of detectable C-peptide [60], and in patients with detectable C-peptide, the proinsulin/C-peptide ratio is increased [60,61]. In agreement with these findings, proinsulin-enriched insulin-poor cells were observed in the pancreas of patients with T1D [60,62,63]. Based on the positive staining for insulin in glucagon+ cells observed in the present study, it can be suggested, that that these calls may contain not only insulin, but also various posttranslational modifications such as proinsulin, C-peptide, and preproinsulin, and may contribute to the plasma proinsulin level in patients with T1D. Taken together, experimental findings showing the switch between β- and α-cell phenotypes and positive staining for insulin in glucagon+ cells allowed researchers to assume that dedifferentiated and/or differentiated β-cells that cannot acquire a mature phenotype can be present among glucagon+ cells of insulin-deficient islets of patients with T1D. Analysis of factors regulating the insulin biosynthesis and maturation of β-cells may be useful for the development of new approaches for diabetes therapy.

Another important issue is the alteration of the phenotype of α-cells in T1D. As shown in several studies, α-cells of insulin-deficient islets of patients with T1D have reduced expression of α-cell specific transcriptional factors ARX and MAFB [64], while expressing some β-cell specific transcriptional factors such as NKX6.1 and PDX1 [29,64]. Moreover, these alterations in the α-cell phenotype are associated with a reduction in glucose-mediated glucagon secretion [64,65,66]. In combination with the impossibility of downregulation of insulin secretion in response to a falling plasma glucose level due to a dramatic loss of β-cells [67,68,69,70], an impaired counterregulatory response, in particular glucagon secretion, results in an increase in the incidence of hypoglycemia in T1D patients during the progression of the disease. Thus, detailed knowledge of the mechanisms underlying the alterations of the phenotype of islet β- and α-cells, as well as understanding the relationships between these islet cell types, may help to develop approaches for the improvement of glucose homeostasis in patients with T1D.

## 5. Conclusions

In the present study, the ability of various antibodies to insulin to detect insulin in the pancreas of persons with T1D was compared. It has been demonstrated that immunoreactivity to insulin in cells of insulin-deficient islets was detectable by monoclonal antibodies that have reported cross-reactivity with proinsulin, and by polyclonal antibodies that may recognize various epitopes located within the A-chain and B-chain of the insulin molecule, and also epitopes within C-peptide. In contrast to previous data showing a low level of insulin in glucagon+ cells of insulin-deficient islets of patients with longstanding T1D, in the present study, immunoreactivity to insulin was observed not only in glucagon+ cells of insulin-deficient islets, but also in the majority of glucagon+ cells of insulin-containing islets of children with recent-onset T1D and nondiabetic persons. Thus, glucagon+ cells may contain insulin and/or proinsulin. The existence of close relationships between the proportion of insulin+ cells and glucagon+/insulin+ cells within the islets was shown using quantitative morphometric analysis. It has been also demonstrated that the formation of insulin-deficient islets could be accompanied not only by the loss of insulin+ β-cells, but also by an increase in the proportion of glucagon+/insulin+ cells. Based on these findings, it was assumed that the plasticity of endocrine cells may contribute to the formation of insulin-deficient islets in T1D. However, future studies are needed to evaluate mechanisms underlying the formation of insulin-deficient islets in T1D and the role of α-cells in insulin secretion and diabetes pathogenesis.

## 6. Limitations

Morphological studies on humans are often performed on autopsy material. Therefore, postmortem changes, fixation, and tissue processing may influence the results. Despite all antibodies applied in the present study exhibiting a similar distribution of the immunoreactivity in all investigated pancreatic samples, the effects of the abovementioned factors cannot be absolutely excluded.

It is well known that many parameters, including individual variations, age-related changes, various physiological conditions, disease states, and medications, etc., have effects on the morphology of the endocrine pancreas. However, these effects cannot be adequately distinguished on the limited number of samples investigated in the present study.

The investigated samples of the pancreas were formalin-fixed paraffin-embedded blocks. This limits the methods that can be used, including the application of molecular genetics approaches. As staining for insulin in cells of insulin-deficient islets was detected by using polyclonal anti-insulin antibodies and monoclonal anti-insulin antibodies that have reported cross-reactivity with proinsulin, and the additional insulin immunoreactivity was mostly co-localized with glucagon in cells of insulin-deficient and insulin-containing islets, it was assumed that glucagon+ cells may contain insulin or other insulin-like proteins (proinsulin, C-peptide). However, the exact structure of the protein interacting with the anti-insulin antibodies in the glucagon+ cells should be further determined, and its transcription and translation needs to be confirmed by biochemical and molecular genetics methods. Despite glucagon+/insulin+ cells being more likely represent α-cells, in the present study, no additional β- or α-cell specific markers were applied to estimate whether the glucagon+ cells expressing insulin were true α-cells, or whether these cells may represent dedifferentiated/transdifferentiated other islet cell types (in particular, β-cells). Therefore, the nature of these cells should also be determined in the future.

On the basis of the presence of immunoreactivity to insulin in glucagon+ cells, and the observed relationships between the proportion of insulin+ and glucagon+/insulin+ cells, it was speculated that the plasticity of endocrine cells may contribute to the formation of insulin-deficient islets in T1D. Investigation of the role of islet cell plasticity in the pathogenesis of diabetes is one of the prospective directions in current diabetology, which is actively being pursued in experimental studies. To evaluate the role of islet cell plasticity in the pathogenesis of diabetes in humans, detailed characterization of different islet cell populations using β- and α-cell specific markers, single-cell RNA sequencing etc. is needed.

## Figures and Tables

**Table 1:** Characteristics of T1D patients and control subjects.

Case	Sex	Age	T1D Duration	Blood Glucose Level	Insulinotherapy	Cause of Death	No. of Blocks Studied
1	Male	3 years 9 month	6 days	23.51 nmol/L	Actrapid (20 U/D)	Diabetic coma complicated by acute pulmonary insufficiency and cardiac decompensation due to acute respiratory viral infection	6
2	Male	13 years 11 month	4 days	33 nmol/L	Actrapid (25 U/D)	Multiple organ failure associated with diabetic coma and ketoacidosis	7
3	Male	27 years	18 years	N/A ^1^	N/A	Anemia due to esophageal variceal hemorrhage	1
4	Male	41 years	26 years	N/A	N/A	Chronic renal insufficiency associated with T1D	1
5	Male	4 years 9 month	Nondiabetic	≤5.5 nmol/L	Not treated	Congenital combined immunodeficiency and bacterial sepsis complicated by anemic brain infarct, disseminated intravascular coagulation, pulmonary insufficiency and cardiac decompensation	1
6	Female	8 years 11 month	Nondiabetic	≤5.5 nmol/L	Not treated	Jungle fever complicated by disseminated intravascular coagulation, hemorrhagic brain edema, cardio-pulmonary insufficiency and cardiac decompensation	1
7	Female	25 years	Nondiabetic	≤5.5 nmol/L	Not treated	Dilated cardiomyopathy complicated by pulmonary edema and cardio-pulmonary insufficiency	1

^1^ Data are not available.

**Table 2:** Characteristics of the primary antibodies used.

Primary Antibodies	Company	Cat#/RRID	Dilution Ranges	Optimal Dilutions
Mouse monoclonal antibodies to insulin, clone K36aC10	Sigma, St. Louis, MO, USA	I2018/RRID:AB_260137	1:1000–1:64,000	1:16,000
Mouse monoclonal antibodies to insulin	Thermo Fisher Scientific Inc., Fremont, CA, USA	MS-1379-P/RRID:AB_62834	1:50–1:500	1:100
Rabbit polyclonal antibodies to insulin	Santa Cruz Biotechnology, Santa Cruz, CA, USA	Sc-9168/ RRID:AB_2126540	1:50–1:200	1:100
Rabbit polyclonal antibodies to insulin	Abcam, Cambridge, UK	Ab63820/RRID:AB_1925116	1:50–1:2000	1:200
Rabbit polyclonal antibodies to glucagon	Thermo Fisher Scientific Inc., Regensburg, Germany	PA5-13442/RRID:AB_2107206	1:50–1:200	1:50
Rabbit polyclonal antibodies to glucagon, clone K79bB10	Sigma, St. Louis, MO, USA	G2654/RRID:AB_259852	1:1000–1:16,000	1:4000
Rabbit polyclonal antibodies to somatostatin	Abcam, Cambrige, UK	Cat#ab103790/RRID:AB_10711731	1:50–1:400	1:50

**Table 3:** Values of the investigated morphometric parameters in insulin-containing and insulin-deficient islets.

Parameter	Insulin-Containing Islets (Control)	Insulin-Containing Islets (T1D)	Insulin-Deficient Islets (T1D)
Islet area, µm^2^	14,233.90 (9719.22–23,035.77) (n = 90) ^1^	22,129.98 (13,898.48–32,860.76) (n = 90)	12,735.23 (81,4840–19,678.74) (n = 90)
Cell density (no. of cells per µm^2^ of islet area)	0.007003 (0.006128–0.007818) (n = 90)	0.008307 (0.007224–0.008815) (n = 90)	0.011385 (0.009767–0.013352) (n = 90)
Proportion of insulin+ cells to total cell no. (%)	48.43750 (43.75000–53.65854) (n = 30)	55.75768 (45.59944–67.14421) (n = 20)	0.00000 (0.00000–0.00000) (n = 40)
Proportion of glucagon+/insulin+ cells to total cell no. (%)	35.01359 (29.26829–41.56627) (n = 30)	26.01351 (18.01948–31.44325) (n = 20)	67.79580 (58.11443–75.43831) (n = 40)
Proportion of glucagon+ to total cell no. (%)	1.847458 (0.000000–2.797203) (n = 30)	1.923785 (1.020408–3.308411) (n = 20)	0.000000 (0.000000–0.439569) (n = 40)
Proportion of somatostatin+ cells to total cell no. (%)	5.96927 (3.21271–8.86587) (n = 60)	5.28891 (3.27808–7.09256) (n = 40)	8.81834 (6.06592–12.88623) (n = 80)
Proportion of insulin+/somatostatin+ cells to total cell no. (%)	0.000000 (0.000000–0.000000) (n = 30)	0.403661 (0.00000–0.756471) (n = 20)	0.000000 (0.000000–0.000000) (n = 40)
Proportion of glucagon+/somatostatin+ cells to total cell no. (%)	0.00000 (0.000000–0.76336) (n = 30)	0.35357 (0.00000–0.73309) (n = 20)	0.94340 (0.000000–0.439569) (n = 40)

^1^ Values are presented as the median and upper and lower quartile values (Me (q1–q3)) and the number of observations (n).

## Data Availability

Data presented in this study are contained within this article and Supplementary Material (Figures S1 and S2, Tables S1–S4). The original microscopy images can be downloaded at https://zenodo.org/records/14230000?token=eyJhbGciOiJIUzUxMiJ9.eyJpZCI6IjYzNjQ0YmU2LTJjMjAtNDI5Zi1hZjI3LTNkMGQyYzRkNjJjMSIsImRhdGEiOnt9LCJyYW5kb20iOiJhMWJhMTkyYzlmMGE0YWUyZjBjNGFiNWU4ZDM5N2U2NiJ9.-KjPf4f_WEY4LerIVD-5YaS3lOLhwnZRUfrEP0unSeiliBE0rzz66BlHcBPXwSK8U8XNVb3-OPmAoxuaXA-zqQ (assessed on 27 November 2024).

## Supplementary Materials

The following supporting information can be downloaded at: https://www.mdpi.com/article/10.3390/life15010125/s1, Figure S1: Intracellular distribution of the immunoreactivity to insulin and glucagon in insulin-containing islets of control subjects, insulin-containing islets of children with recent-onset T1D, and insulin-deficient islets of T1D patients; Figure S2: Statistical analysis of the distribution of insulin+/somatostatin+ and glucagon+/somatostatin+ cells in insulin-containing islets of control subjects, insulin-containing islets of children with recent-onset T1D, and insulin-deficient islets of T1D patients; Table S1: Quantification of insulin-containing islets in children with recent-onset T1D; Table S2: Quantification of insulin+, glucagon+/insulin+, and glucagon+ cells in insulin-containing islets of control subjects, insulin-containing islets of children with recent-onset T1D, and insulin-deficient islets of T1D patients; Table S3: Quantification of insulin+, somatostatin+, and insulin+/somatostatin+ cells in insulin-containing islets of control subjects, insulin-containing islets of children with recent-onset T1D, and insulin-deficient islets of T1D patients; Table S4: Quantification of glucagon+, somatostatin+, and glucagon+/somatostatin+ cells in insulin-containing islets of control subjects, insulin-containing islets of children with recent-onset T1D, and insulin-deficient islets of T1D patients.
