# Supplementary material for: Detection of Insulin in Insulin-Deficient Islets of Patients with Type 1 Diabetes
Source: Life (Basel). 2025 Jan 19;15(1):125. doi: 10.3390/life15010125 (PMC11766825; doi:10.3390/life15010125)
Supplement: Supplementary file 1 [file life-15-00125-s001.zip › Table S1.pdf]

Table S1. Quantification of insulin-containing islets in children with recent-onset T1D.

Case No.	No. of block	Location in the pancreas	Section area (μm²)	Total No. of islets	No. of insulin-containing islets	Percentage of insulin-containing islets
Case 1	1	Tail	57500000	206	0	0%
	2	Body	98400000	203	12	5.91%
	3	Body	132000000	291	29	9.97%
	4	Body	120000000	229	37	16.16%
	5	Body	110000000	208	9	4.33%
	6	Head	134000000	159	68	42.77%
			**Total:**	**1296**	**155**	**11.96%**
Case 2	1	Tail	57020000	213	0	0%
	2	Tail	84800000	221	6	2.71%
	3	body	75500000	163	12	7.36%
	4	body	68000000	143	29	20.28%
	5	body	105000000	300	20	6.67%
	6	body	66200000	138	32	23.19%
	7	head	87300000	133	33	24.81%
			**Total:**	**1311**	**132**	**10.07%**
