# Supplementary figures and images for: Detection of Insulin in Insulin-Deficient Islets of Patients with Type 1 Diabetes
Source: Life (Basel). 2025 Jan 19;15(1):125. doi: 10.3390/life15010125 (PMC11766825; doi:10.3390/life15010125)

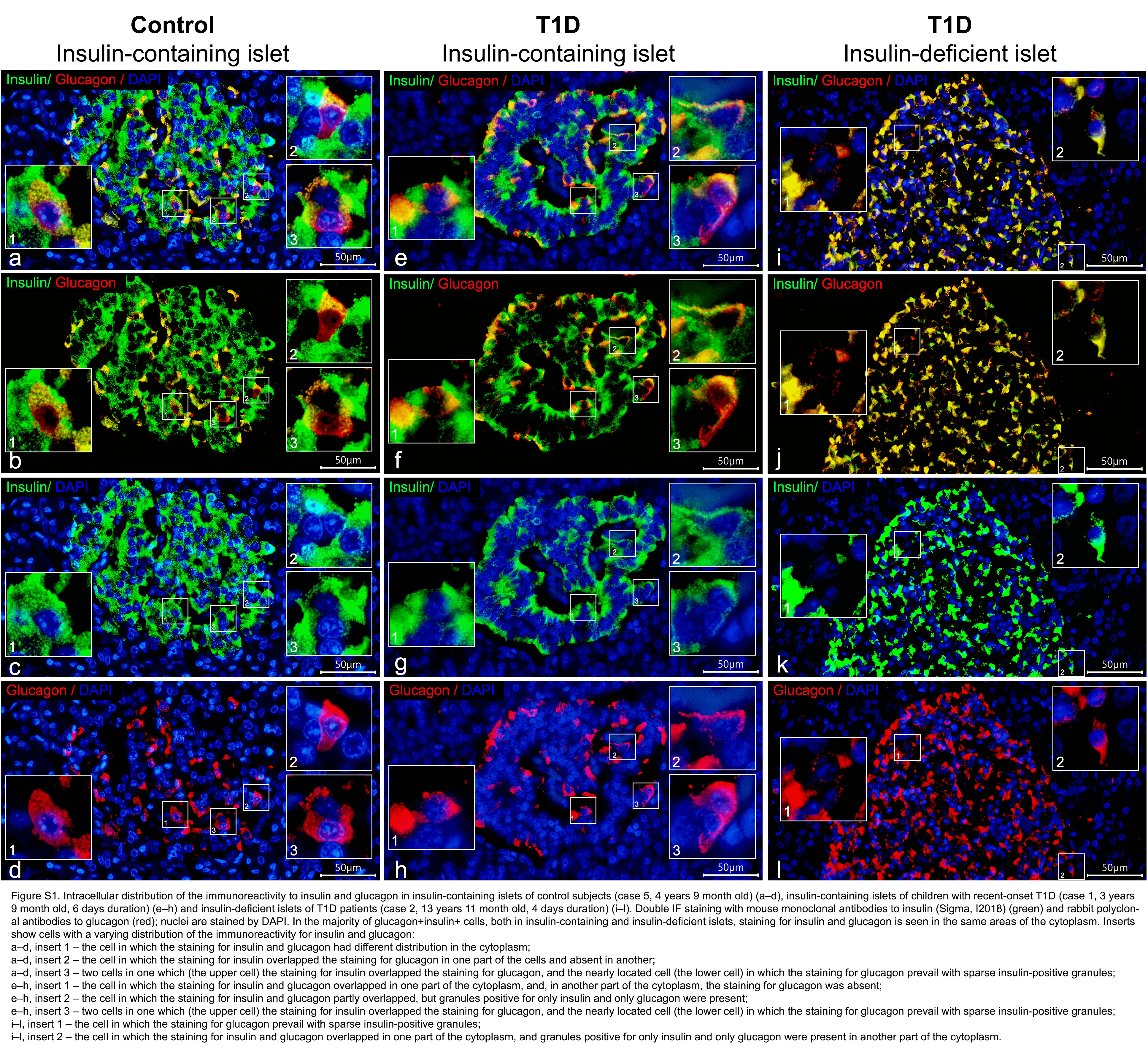

Supplement: Supplementary file 1 [file life-15-00125-s001.zip › Figure S1.jpg]

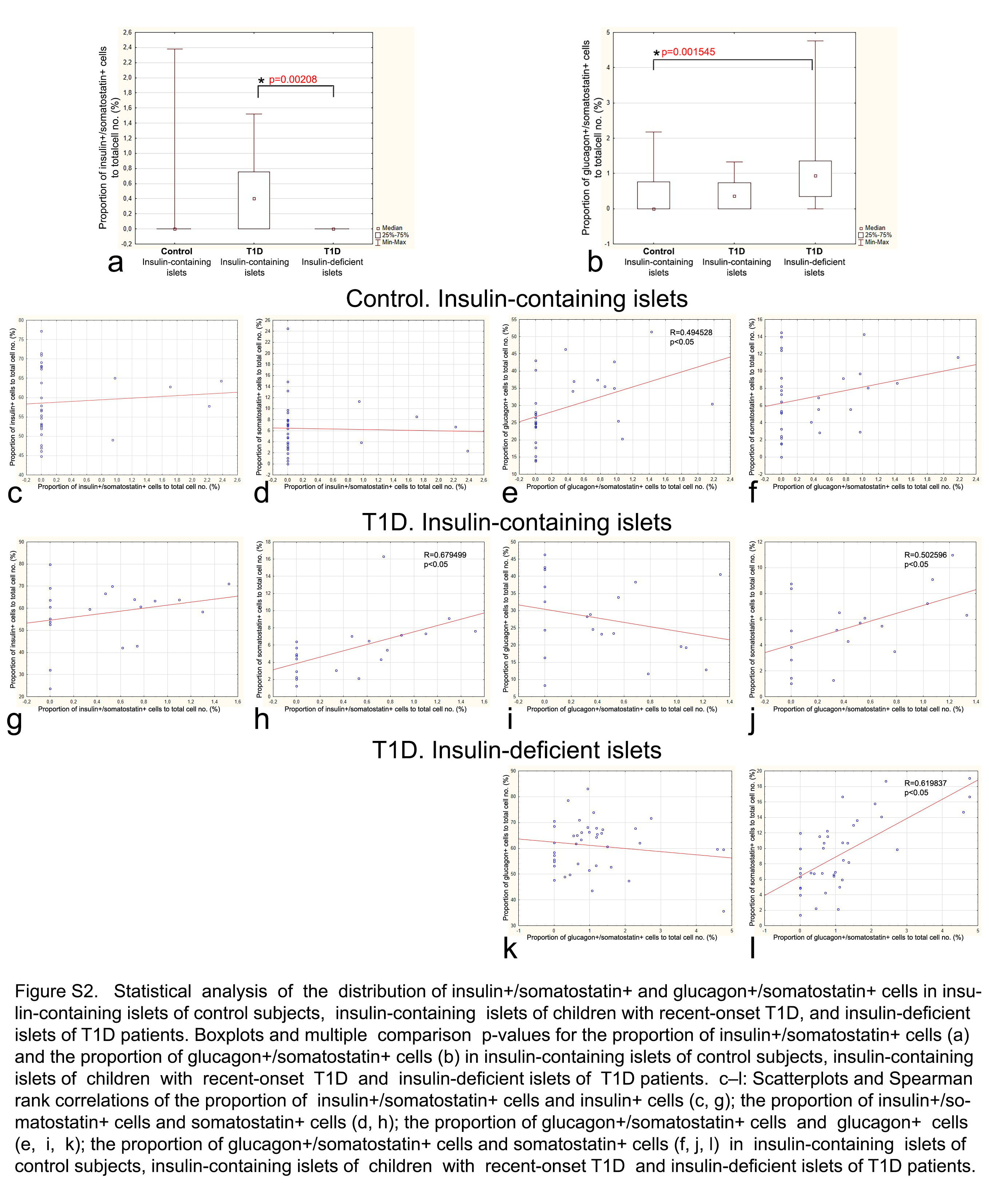

Supplement: Supplementary file 1 [file life-15-00125-s001.zip › Figure S2.jpg]
